# Grassroots stakeholders’ perception of participation in the Medium-Term Development Plan of District Assemblies in Ghana: The case of Sawla-Tuna-Kalba District

**DOI:** 10.1016/j.heliyon.2023.e19178

**Published:** 2023-08-16

**Authors:** Asaah Sumaila Mohammed, Moses Naiim Fuseini, Kuupiel Cuthbert Baba

**Affiliations:** a Department of Environmental Science, CK Tedam University of Technology and Applied Science, Navrongo, Ghana; b Department of Public Policy and Management, SD Dombo University of Business and Integrated Development Studies, Wa, Ghana; c Dr Hilla Limann Technical University, Wa, Ghana

**Keywords:** Participation, Grassroots, Stakeholder, Medium-Term Development Plan, Local government, Ghana

## Abstract

Following Ghana's decentralisation policy, the District Assemblies, in consultation with communities, are required to prepare and implement Medium-Term Development Plans [MTDPs] to ensure the overall development of their respective jurisdictions. However, little consideration has been given to the participation of grassroots stakeholders in the development of MTDPs. Consequently, this study investigates the participation of grassroots stakeholders in developing MTDPs. A cross-sectional survey involving 139 respondents was deployed. The data were gathered using a questionnaire and an interview guide, and it was analyzed using descriptive statistics, Spearman's Rank Correlation, and thematic analysis. Results indicated that grassroots stakeholders were aware of their right to participate in the preparation of MTDPs. As such, they actively participated in the process that led to developing such plans addressing community needs. Additionally, awareness of MTDP, involvement, level of influence, satisfaction with the preparation of MTDPs, satisfaction with the quality of participation in the area council, level of confidence in the preparation of MTDP, representation adequacy, and capture of community needs had statistically significant associations at the 1% level with the associations being positive. Nonetheless, the implementation of MTDPs is jeopardised by limited funding, stakeholder commitment, and human capacity. To ensure the smooth implementation of the MTDPs, the Assembly must intensify its revenue mobilisation efforts, depoliticise the MTDP process, and build staff capacity on the involvement of the grassroots stakeholders.

## 1 Introduction

Early theories and practises of development focused on top-down diffusion, which exacerbated inequality and occasionally resulted in resource waste [1,2]. As a result, development was approached top-down and from a centralised perspective [3]. However, the top-down approaches failed to deliver the needed development to the people, especially at the local [community] level, because of the centralisation of decision-making and resources [4]. Since then, there has been a commitment to decentralise local governments' political, financial and decision-making authority to provide public services and adequately meet citizens' diverse needs in their catchment areas, which is possible if there is grassroots participation [3,5,6]. This commitment is necessary to address the development shortfall associated with top-down approaches. According to Odo [7], local government is the people's government at the local level. It should be considered as involving them in its operations to capture and address their needs and aspirations adequately. Therefore, local governments get their sense of grassroots democracy through the involvement of the populace in the decision-making and administration of their provinces. The failure of conventional development thoughts and actions to improve people's living situations led to an alternative development paradigm known as participatory development in the 1980s [8,9]. Participatory development principles are based on the rights-based approach, emphasising stakeholder involvement in local development processes [2,10].

Participation of stakeholders in the planning and implementation of development at the local government level is essential as it fosters the satisfaction of felt needs, ownership, and sustainability of such initiatives [[11], [12], [13], [14]]. It also ensures that everyone is responsible for the budget and service delivery [15]. As a result, some African nations, including South Africa, Kenya, Tunisia, Zimbabwe, and Uganda, have enacted laws to guarantee local people's involvement in the development planning processes [12,16]. Despite this, these countries stagnate in their development efforts, especially locally. This situation is partly attributed to grassroots stakeholders' minimal or non-existent involvement in formulating and implementing development plans [12]. Accordingly, Kinyata and Abiodun [12] have stated that the bottom-up development method should bring the public closer to the government through involvement in developing and implementing development projects.

Ghana employs a decentralisation system as part of the public administration structure, as stipulated by the Local Government Act [Act 936] of 2016, the 1992 Constitution and earlier, Local Government Act 462. District Assemblies are designated as the highest political, legislative, budgetary, and planning authority at the local level by the Local Government Act. The system has a three-tier Municipal and District Assembly and a four-tier Metropolitan Assembly [17]. The Local Government Act of 2016 serves as the cornerstone for participatory governance at the local level [Act 936]. The Act's Sections 40–48 provide for participatory governance at the local level, including the freedom for residents to take part in District Assembly activities like project design and execution. To ensure the general development of their particular districts, District Assemblies must create and carry out their development plans. To accomplish this, per the National Development Planning System Act of 1994 [Act 480], the National Development Planning Commission offers Medium-Term Development Plan [MTDP] preparation guidelines for the District Assemblies [18]. The District Assemblies have been creating MTDPs since 1996. Whether or not grassroots stakeholders participate in the MTDPs, as required by the local government statute and the National Development Planning Commission [NDPC], is a crucial question that needs to be addressed.

Although authors have done studies on local governance, including Kobani [19], Nwogwugwu and Odedina [20], Bakare [10], and Damayanti and Syarifuddin [3], they tended to focus more on elections than on local government development planning. The work of Rusnaini [21], which focused on Indonesia's Medium-Term Local Development Plan, appears to be the exception because it discussed local-level stakeholders'' participation. Though some research in Ghana has focused on stakeholders' involvement in the MTDPs of the District Assemblies [18,[22], [23], [24]], such studies are incomplete. Ofosu and Ntiamoah [24], for instance, very briefly discussed the monitoring and evaluation component of the MTDP, whereas Mensah [18] and Agbenyo et al. [23] concentrated on implementation challenges. Abubakari and Ayuune [22] approached MTDP as a peripheral issue earlier. The evidence suggests some gaps. The knowledge gap is that most prior studies on participation in MTDPs needed to be more comprehensive and/or treat the issue as peripheral—consequently, the need to investigate grassroots stakeholders' participation in the MTDP of District Assemblies in Ghana. Specifically, the objectives are to: (1) examine the extent of grassroots stakeholders' participation in the design of MTDPs, (2) analyse the effects of grassroots stakeholders' participation in the MTDPs and (3) explore the challenges to the implementation of the MTDPs. Since resources are scarce, the focus of this study is imperative to policymakers because it would reveal the extent to which the stakeholders are engaged in the design of the MTDPs and whether such efforts translate into the provision of development initiatives that meet their felt needs. Besides that, the unique contribution of this study is that it pays maximum attention to grassroots stakeholders' participation in MTDPs by looking at the extent of participation, its effects and the challenges thereof, which previously gained little attention from researchers.

## 2 Literature review

### 2.1 Conceptual review

This segment reviews the fundamental concepts [i.e., local governance, participation, grassroots participation and MTDP] that drive the study. The first of such concepts is local governance. Local stakeholder interaction in setting the local agenda, managing resources, and carrying out development initiatives is called local governance [25]. Abdul-Razak, Prince, and Eliasu [26] contend that local governance can be viewed as distributing power and responsibility among all parties, including local interest groups, and ensuring adequate representation of viewpoints. Therefore, local government entails transferring power to the local areas so they can take charge of their development. The Assembly serves as the first tier of local government in Ghana. The General Assembly, the Executive Committee and Subcommittees, the Coordinating Directorate, and the Decentralised Departments comprise a District Assembly framework [27]. Local governance creates space for participation. Participation is the process through which a person plays a part in the political life of his society and gets the chance to decide what the society's shared goals are and how to achieve them best [28]. "Participation in development” is defined as "beneficiaries' involvement in all stages of the process of development, including decision-making, execution, monitoring, evaluation, and management of their development programmes,” according to Jamadar [29].

On the other hand, the United Nations [30] conceives grassroots involvement as the establishment of opportunities to allow all people of a community to actively contribute to and influence the development process and share equally in the benefits of development. Participation at the local level and involving citizens at the grassroots in governmental affairs are also included in the definition of “grassroots participation” [10]. The democratic approaches to public policy, community planning, and development that presume people have the right to make decisions that impact their lives are the foundation of grassroots participation [31]. Therefore, community involvement fosters local-level development by raising residents' living standards [7]. According to Fu and Distelhorst [32], there are two types of grassroots participation: institutionalised and controversial. Institutionalised involvement uses state-sanctioned channels, including municipal elections, government hotlines and mailboxes, and courts, to affect policy, address and resolve grievances, or decide on cases involving the public and governmental bodies [32]. Contrarily, disruptive engagement comprises making a symbolic or political message through protests, petitions, strikes, and the formation of illegitimate organisations [32]. The United Nations' [30] conceptualisation of grassroots participation and Jamadar's [29] definition of participation apply to this study.

In terms of planning, it denotes “a continuous process which involves decisions, or choices, about alternative ways of using available resources, to achieve particular goals in the future [33, p. 3]. Planning is crucial because resources are limited in proportion to human demands, making efficient and effective use a constant requirement. The creation of MTDPs is one of the routes. According to Krause and Kim [34], MTDPs are plans with durations ranging from three to seven years and typically include objectives, predictions, and policy measures. As a tool for policymaking to ensure the overall development of their operational areas, the National Development Planning Commission instructs District Assemblies in Ghana on creating and implementing MTDPs. These MTDPs [i.e., 1996–2000, 2002–2004, 2006–2009, 2010–2013, 2014–2017, 2018–2021, and 2022–2025] typically last between one and five years. MTDPs are thus an obligation of the District Assemblies in Ghana. This is done to ensure that development reflects the felt needs of the people [those at the grassroots] within the catchment areas of the District Assemblies.

### 2.2 Grassroots stakeholders’ participation in local governance

This section presents an empirical review of studies on participation in local governance. A study by Abubakari and Ayuune [22] on women's involvement in local government in the Ghanaian municipality of Kassena Nankana found a deficient level of participation among women. In addition, fewer chances were established for women's engagement, while obstacles such as an unfavourable political environment and a low level of education limited it. According to Ofosu and Ntiamoah's [24] analysis of the Kwahu West Municipal Assembly, community involvement in development projects has the highest correlation coefficient, followed by participatory local governance and monitoring. Mohammed [35] found that Ghana's mechanisms for encouraging local government participation must be revised. Due to gender-insensitive decentralisation policies, lack of socioeconomic resources, low educational attainment, and cultural customs, only the privileged males participate, leaving out women, the weak, and people living in rural areas. According to a similar study by Agbenyo et al. [36], participatory monitoring and evaluation give the stakeholders in Ghana's Upper West Region influence over project execution, allowing them to initiate corrective actions to address discrepancies. In any case, women and young people were partially involved in the process. Furthermore, some beneficiaries needed help due to their lack of capacity and understanding of their tasks and their sporadic involvement. According to Agbenyo et al.'s [23] study, which focused on sub-district structures' participation in the formulation of the Nadowli-Kaleo District MTDP, it was found that low levels of commitment by stakeholders and ineffective teamwork were some of the implementation challenges of MTDPs. Mensah's [18] research of six district assemblies in Ghana revealed that MTDP implementation issues included poor institutional frameworks, insufficient human and financial resources for the District Assemblies, chieftaincy and property disputes, low stakeholder commitment, and inefficient cooperation.

Similarly, Nwogwugwu and Odedina's [20] study in Ghana and Nigeria revealed that the political participation of grassroots women substantially impacts poverty in both countries. According to Odo [7], the obstacles to community involvement in development include a lack of qualified politicians, overzealous local politics, corruption, a lack of community participation in the development process, a misalignment of priorities, a lack of autonomy, and a weak financial foundation for local governments. Related conclusions were reached by Bakare [10], who found that in Osun State, Nigeria, poor government accommodation and responsiveness, lack of information about government initiatives, distrust of political officeholders, and low levels of public awareness of their roles in governance are the leading causes of the state's low levels of grassroots participation. In their 2020 study, Kinyata and Abiodun examined how much community involvement affects the viability and sustainability of development projects in Africa. It was found throughout the 1980s and 1990s that development aid to Africa, including Uganda, only benefited some countries since the bottom-up approach was not used. Mohammad [37] showed that while elected Union Parishad members in Bangladesh participate equally in planning development projects, the public's involvement in those projects' planning stages is minimal. Creating Project Implementation Committees is merely an act of formality, during which neither the members are sufficiently consulted nor informed of the projects' progress in implementation. As a result, participation in project implementation committees is minimal and frequently artificial. Community involvement in project planning is as low as 7% but increases to 24% during implementation. The perception that development projects are typically non-participatory, however, is ubiquitous.

As stated by Rusnaini [21], participation in the Medium-Term Local Development Plan in Indonesia was on placation, which means a higher degree of tokenism because the ground rules permit citizens to offer advice but preserve the powerholders' ongoing authority to make decisions—limitations on the public consultation forum's time for participation. In a study conducted in Indonesia, Damayanti and Syarifuddin [3] found that participation in development planning discussion forums was limited to individuals connected to the government, leaving out most of the public. Furthermore, rural development methods fail to address community demands because participatory planning does not continuously involve the community in the development process. In their study, Abiona and Bello [38] examined the sustainability of development programmes in Nigeria and the participation of the general public in the decision-making process. The findings demonstrated a strong correlation between grassroots involvement in development programmes, the decision-making process, and the durability of those programmes. In addition, sustainability could have been improved by political instability, issues with leadership, racial tensions, inadequate money, and poor accountability. In Nigeria's Etche Local Government Area, Kobani's [19] study found that the general public needs a better understanding of community development and its goals. It also showed the lack of local community participation in needs assessment, planning, implementation, monitoring, and evaluation tasks. According to Mubita et al. [39], participation can also enable the incorporation of local knowledge, skills, and resources into the design of interventions; ensure that projects and programmes are responsive to people's needs, help achieve sustainability, and support the dismantling of dependency mentalities. Critics say participation does not make residents more powerful because participatory approaches must change or challenge the bureaucratic, centralised, and administrative systems that make decisions and distribute resources.

### 2.3 Theoretical perspectives

**Fig. 1:**
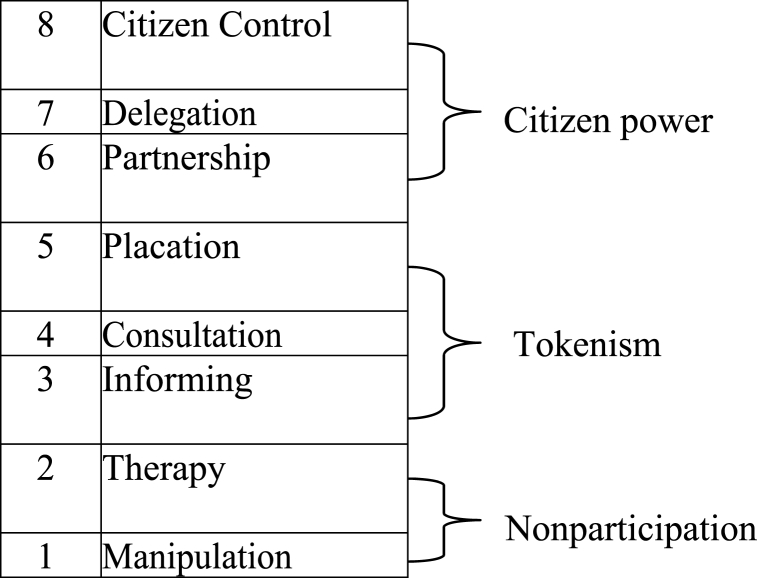
Degrees of citizen participation.

Source: Adapted from Arnstein [40].

The ladder of citizen participation , Pretty's theory of participation, and rational choice theory underpin this study. The ladder of citizen participation theory explains how citizens can take part in a particular activity [40]. Collins and Ison [41] state that the primary objective of the ladder of citizen participation is to distinguish between the many levels of participation and the power associated with each level in ascending order of priority [Fig. 1]. Manipulation and Therapy are at the bottom of the ladder and refer to levels of “non-participation” that some have created to stand in for actual participation [40]. Their true goal is to enable those in positions of power to “teach” or “cure” the participants rather than to allow them to participate in programme development or execution [40]. As a result, this kind of engagement was referred to as passive or pseudo-participation by Midgley et al. [42]. The progression of rungs 3 [information] and 4 [consultation] reaches degrees of “tokenism” that enable the underprivileged to be heard and have a voice [40]. Citizens can hear and be heard when they offer themselves full involvement. However, given these circumstances, they cannot guarantee that the power brokers will pay attention to their opinions. When involvement is limited to these levels, there is no follow-through and no “muscle”; thus, there is no guarantee that the status quo will change. It is because the ground rules permit have-nots to offer counsel, but the power holders still have the right to make decisions. As such, placation is essentially a higher kind of tokenism [40].

There are various tiers of citizen power with varying degrees of influence on decisions [40]. Citizens can bargain and make trade-offs with traditional power brokers by entering into a (6) Partnership. The highest levels, (7) Delegated Power and (8) Citizen Control, provide have-not people most seats at the table or full management authority. Midgley et al. [42] state that active or genuine engagement correlates with citizen power. In a democracy, the people actively choose the policies that affect them. Academics, policymakers, and development practitioners have found Arnstein's Ladder to be a valuable framework for evaluating the success of stakeholder participation in development processes [41]. As a result, the theory served as a helpful lens for analysing the involvement of the MTDPs in the District Assemblies. Despite the theory's applicability, detractors contend that it ignores obstacles to obtaining actual levels of involvement and that sometimes citizens' participation in decision-making is not about gaining power [41,43]. The identified weakness in this theory was addressed as it was complemented by Pretty's theory of participation and rational choice theory which brings to the fore the motivations of citizens and authorities in the participation processes.

Pretty's typology of participation focuses on users of participatory approaches and the spectrum of a shift in control from authorities to the citizens [44]. It describes the motivation for which participants are involved at each level. Pretty's theory differs from Arnstein's in this aspect of the motivation of participation of users. However, Pretty's typology, like Arnstein's ladder, represents a progression from weak forms of participation [manipulative & passive] to better forms [interactive & self-mobilisation] [Table 1]. The critique of this theory is that it needs to adequately consider the contextual factors that can influence the effectiveness and appropriateness of different participation approaches and that the typology only sometimes relies on a solid empirical evidence base to support its categorisation. This limitation is addressed by complementing it with rational choice theory, which focuses on rational beings' decisions [45]. Despite the critiques of the theory, it is relevant in explaining the motivation for involving people in the District Assemblies' MTDPs.

**Table 1:** Pretty's typology of participation.

Type of Participation	Features
Manipulative Participation	Pretence, with nominated representatives having no legitimacy or power
Passive Participation	Unilateral announcements without listening to people's responses
Participation by Consultation	External agents define problems and information-gathering processes, and so control analysis
Participation in Material Incentives	People participate by contributing resources [labour] in return for material incentives
Functional Participation	External agencies encourage participation to meet predetermined objectives
Interactive Participation	People participate [as a right] in joint analysis, development of action plans and formation or strengthening of local institutions
Self-Mobilisation	People take initiatives independently of external institutions to change systems

For the rational choice theory, it contends that participation is entirely focused on the individual and the decisions he or she makes [45]. According to the theory, people's decisions reflect the costs and advantages of their decision-making situations, leading to civic involvement [45]. People are supposed to choose their levels of engagement in light of these costs and rewards while also being impacted by social norms and views about the responsibilities and rights of citizenship. As per Uhlaner [47], the defining feature of the rational actor approach to explaining behaviour is the idea that people have preferences and act in ways that further those preferences. A focus on people's incentives is brought to studying involvement by rational actor models [48]. For Downs [49], people will only engage in an activity if the advantages outweigh the drawbacks. According to the rational choice theory, political decision-making results from calculating the costs and benefits [45]. In the context of the rational choice theory of participation, Nie, Junn, and Stehlik-Barry [50] contend that education helps people become better decision-makers. According to Uhlaner [47], the theory's weakness is that despite the rational actor models' cogent explanation of political action motivations, they need to improve their treatment of the community in which political action takes place and their inability to predict future behaviour. This weakness is addressed by the fact that Pretty's theory of participation focuses on a shift in control from the authorities to citizens, which grants them true power in decision-making. Notwithstanding its flaws, it helps explain why people participate in the District Assemblies' MTDPs.

## 3 Methodology

### 3.1 Study setting

**Fig. 2:**
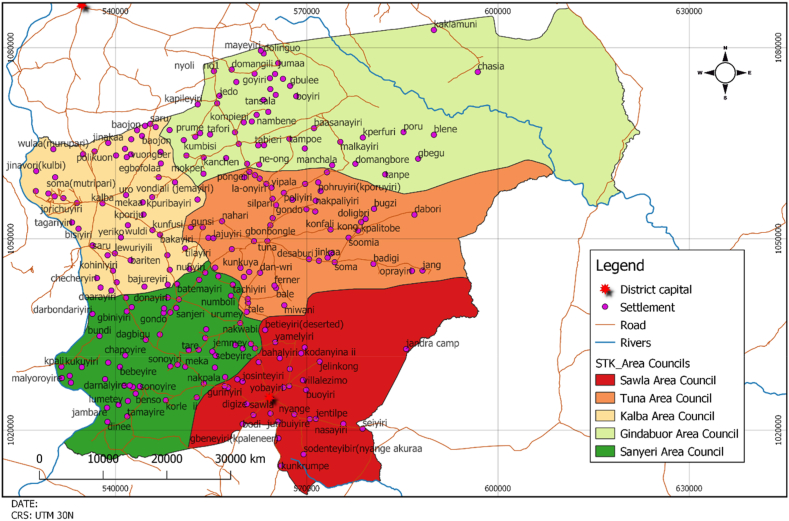
Map of Sawla-Tuna-Kalba district.

Source: Adopted from Sawla-Tuna-Kalba District Assembly [53], p. 8]

Sawla Tuna Kalba District is found in the western part of the Savannah Region. It shares common boundaries with Wa West District and Wa East District to the North, Bole District to the South, West Gonja District to the East, La Cote d' Ivoire, and Burkina Faso to the West [Fig. 2] [51]. The Sawla-Tuna-Kalba District has 33 Electoral Areas, one Town Council, five Area Councils [i.e., Sawla Town Council, Tuna Area Council, Kalba Area Council, Gindabour Area Council and Senyeri Area Council] and 165 Unit Committees. Therefore, the General Assembly of the District comprises 41 Assembly Persons, 33 elected and 14 Government Appointees. Except for Kong-Wura, who is enskinned by Yagbong-Wura, the overlord of Gonja traditional territory, the district contains seven divisional chiefs directly enskinned by Bole-Wura. Gonjas, Vagla, Brifo and Dagaaba are the major ethnic groups in the district, but the chiefs are Gonjas because they are the indigenes and owners of the land. The district has civil society organisations, including Frontiers Mission Network, Care for Deprived Communities, Centre for Active Learning and Integrated Development, Tuna Women Development Project, and NASCO Feeding Minds. As of 2021, the district had a population of 112,664, comprising 59,660 females [52]. Also, 90,133 of its population live in rural areas suggesting it is a rural district.

The district has a poverty incidence ranging from 60 to 66.9%, with a poverty depth ranging from 30 to 34.9% [54]. About 80.1% of households in the district are engaged in agriculture. The road network to the various communities and the main commercial centres is not motorable during the rainy season. The only tarred road that runs through this district from the Upper West region to the South is 125 km. The district has 16 health facilities [one polyclinic, three clinics and 12 operational Community-based Health Planning and Services [CHPS]. The Sawla-Tuna-Kalba District currently has 188 basic schools distributed across eight educational circuits. These comprise 61 nursery/kindergartens, 94 primary schools and 33 junior high schools. The district also has only three senior high schools and one TVET. In terms of road infrastructure, most of the roads are feeder roads and are untarred.

### 3.2 Study design

As regards the study design, the study used a cross-sectional study design. A cross-sectional study design is best suited to finding out the prevalence of a phenomenon or problem by taking a cross-section of the population [55]. The design is suitable for obtaining a general picture as it appears during the study period [55]. The cross-sectional study design is appropriate for this study because it allowed for collecting data from respondents and participants at one point in time on participation in MTDPs, the effects of the participation and challenges associated with the MTDP implementation.

### 3.3 Sample and sampling procedure

The study population comprised Unit Committee Members, Area Council Members, Assembly members, District Planning Coordinating Unit [DPCU] members, and some influential residents in the Sawla-Tuna-Kalba District. A sample size of 133 was calculated using the following parameters: Confidence Level = 75%, Margin of Error = 5%, and population proportion = 50% [https://www.calculator.net/sample-size-calculator.html?type=1&cl=75&ci= 5&pp=50 &ps=&x =40&y=20]. To account for attrition, authors such as Fernandez et al. [56] argue that a proportion of the sample size should be calculated and added to the sample. In this case, 4.5% of 133 is about 6, which is added to obtain the sample size of 139. The sample was selected using stratified random sampling, and the stratification was because the population was heterogeneous. It was to ensure that each category was represented in the sample. The sample distribution was non-proportional [Table 2]. The key informants were two from the District Planning Coordinating Unit. They were selected using purposive sampling. Purposive sampling was used because they have adequate knowledge about citizens participation in the preparation of the MTDPs of the Assembly.

**Table 2:** Sample size distribution.

Respondent Category	Sample
Unit Committee Members	80
Area Council Members	10
Assembly Members	15
Influential residents	34
Total	139

### 3.4 Methodology for searching for studies

#### 3.4.1 Eligibility criteria

To be considered for inclusion in the review, papers should have met at least one of these criteria: have addressed participation, focused on grassroots participation, concentrated on MTDPs, touched on participation in local governance, and discussed theories of participation. The included papers were peer-reviewed journal articles, thesis and reports published from 1959 to 2021 (with the majority from 2015 to 2021), written in English, involving human participants and secondary data. Quantitative, qualitative, and mixed methods studies were included to examine various facets of measuring participation in the MTDP. No submissions were accepted if they focused on general planning or the abstract or full texts could not be accessed.

#### 3.4.2 Information sources, search and selection of sources of evidence

**Fig. 3:**
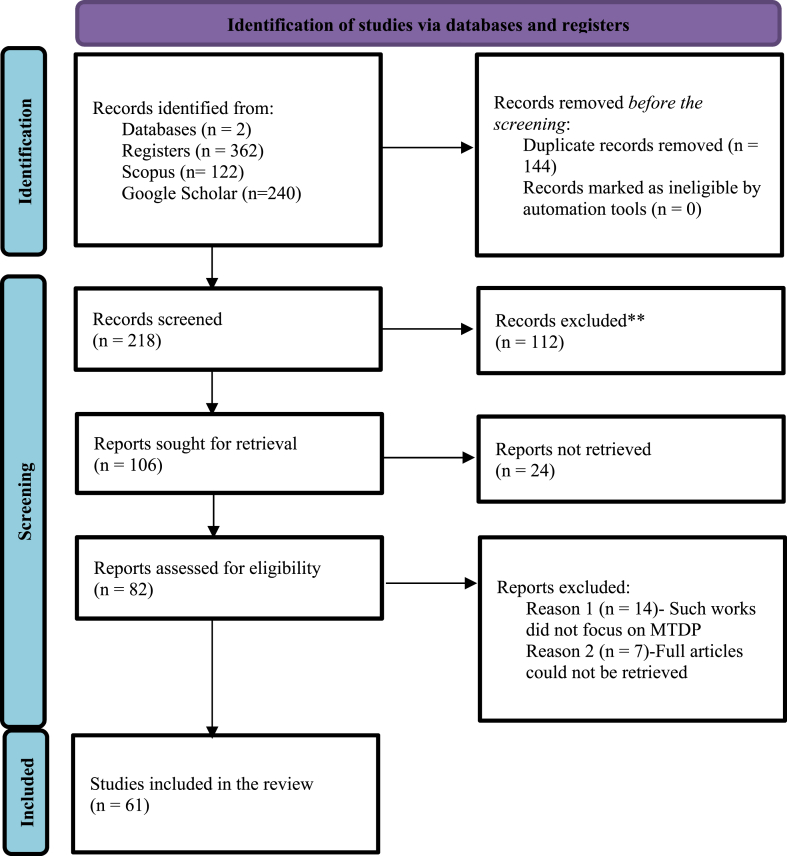
PRISMA flow chart.

Source: Adapted from Page et al. [58].

From January 2022 to June 2023, the following bibliographic databases were searched to identify potentially relevant documents: Google Scholar and Scopus. An experienced librarian at SD Dombo University of Business and Integrated Development Studies drafted the search strategies and refined through team discussion. This article follows the PRISMA-ScR Checklist [Fig. 3] review protocols and registration developed by Tricco et al. [57]. The Google Scholar database search returned 240 results, while Scopus gave 122. Using the inclusion and exclusion criteria, the two researchers screened the titles and abstracts of the studies identified in the initial search. As defined above, it excluded 144 studies due to duplication and predetermined conditions. Following this, a full-text review of potentially relevant articles reveals 61 relevant studies. Disagreements among the reviewers were discussed, and a consensus was reached regarding the final selection. The reference lists of the included articles were examined for primary studies that the search strategy might have missed. Following a full-text analysis, data were extracted from each of the selected studies. These studies examined the definition of grassroots, participation, MTDPs and local governance; Pretty's theory of participation, and rational choice theory; participation in the MTDPs design; effects of participation in the MTDPs; and challenges to implementing the MTDPs.

#### 3.4.3 Data charting process and synthesis of results

Three reviewers developed a data-charting template to determine which variables to extract. In an iterative process, the three reviewers independently charted the data, discussed the results, and continuously updated the data-charting form. The data-charting form included the names of authors; year of publication; study title; study objective; study location; definition of grassroots, participation and local governance; ladder of citizen participation, Pretty's theory of participation and rational choice theory; participation in the MTDPs design; effects of participation in the MTDPs; and challenges to the implementation of the MTDPs. Regarding synthesising the results, the detailed search results were compiled and summarised using texts and a figure. The scope of the study determined the themes. A change was made to the data reporting scheme based on the findings by adhering to the PRISMA-ScR criteria [57].

### 3.5 Instrument, data collection and analysis

A questionnaire and an interview guide were used to collect the data. The researchers created the questionnaire. It included respondent biodata, participation level, participation impacts, and implementation obstacles for the MTDP. In the survey, both closed-ended and open-ended questions were included in the instrument. Most items were measured on a four-point Likert scale, with “highly aware” and “not aware” as the extremes. The researchers also designed the interview guide, covering issues about awareness, participation, the effects of participation, and obstacles to MTDP implementation. Data were gathered from June 1 to June 30, 2022. The questionnaire was pre-tested on 20 respondents with similar backgrounds in the West Gonja Municipality from April 2 to April 6, 2022. It was done to ensure that the questions passed the validity tests for the face, content, and construct and to allow for the clarification of any ambiguities in the questions before the actual administration. The SD Dombo University of Business and Integrated Development Studies Research Ethics Review Board [RERB] was consulted for ethical approval. It ensured that the study complied with all ethical requirements to protect the participants. The RERB report found no risks associated with the respondents' participation in the study. The questionnaire also contained a statement requesting the respondents' informed consent and describing the purpose of the study, assuring them that the study would not be injurious to them, as well as how confidentiality and anonymity would be maintained. The administration of each questionnaire took approximately 30 min while that of the interview guide was 40min.

Before the analysis, the quantitative data were cleaned and imported into SPSS version 24. Descriptive statistics and Spearman's rank correlation were used to analyse the data. Thematic analysis was performed manually on the qualitative data using an inductive methodology. Per the inductive method, emerging themes or codes are typically associated with the data themselves [59]. After codes were discovered and refined and there were no new codes, they were categorised by topic and discussed [60]. The raw data revealed three themes: limited funding, stakeholders' commitment, and capacity.

## 4 Results and discussion

### 4.1 Socio-demographical data of respondents

Sex, age and educational status were this study's respondents' background variables [Table 3]. Regarding the sex of the respondents, most were males [93.5%], whereas, in terms of age, most [62.6%] of them were within the age brackets of 21–40. The finding that most of the respondents were males is consistent with those reported by Adatuu and Apusigah [61], that males are usually dominant in participation in local governance. To this end, Mohammed [35] has argued that this situation occurs in Ghana's mechanisms for encouraging local government participation because they are insufficient and appear to be gender neutral. It indicates that women's issues may not be prioritised in developing the MTDPs since only a few participate. Concerning educational status, most respondents had formal education, with most [54%] completing tertiary education. According to Ghana Statistical Service [52], about 66% of the population in the Savannah Region is literate. It suggests that the respondents have developed human capital and can contribute their quota during engagement in the MTDP development. Similarly, Nie et al. [50] maintain that education helps people become better decision-makers. As such, the respondents' contributions to the design of the MTDPs would be significant.

**Table 3:** Biodata of respondents.

Variable	Frequency	Percent
*Sex*		
Male	130	93.5
Female	9	6.5
*Age*		
21–40	87	62.6
41–60	38	27.3
61+	14	10.1
*Educational Status*		
No Education	18	12.9
Basic Education	16	11.5
Secondary Education	30	21.6
Tertiary Education	75	54.0

### 4.2 Participation in the MTDPs design

**Fig. 4:**
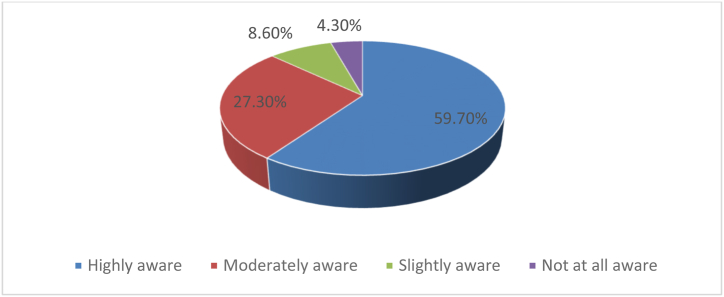
Awareness level of awareness in the preparation of the MTDP.

To ensure that development is sustainable and reflects the needs of the people, the beneficiaries must be involved [2,12], as this promotes a bottom-up approach to development [3,12]. According to rational choice theory, for stakeholders to participate in the process, they must first be aware of the opportunity before exploiting it [45,50]. Consequently, it was essential to determine the extent to which citizens at the grassroots level were aware of their right to participate in the design of their district's MTDPs [Fig. 4]. Most respondents [59.7%] were highly aware of their right to participate in developing their district's MTDPs, as indicated by the survey results. This level of awareness is anticipated to result in actual participation in the process. Evidence from the key informant interviews also suggested that many of the district's residents were aware of their right to participate. For example, a key informant from the Sawla-Tuna-Kalba District Assembly [June 30, 2022] pointed out that:

*In my interactions with some of the residents of our districts, I observed that while some did not know, quite a number of them seemed to know that they had the right to participate in MTDP.*

The quotation illustrates that some citizens are aware of their right to participate in the development of the MTDP of the district. Bakare [10] found that in Osun, Nigeria, there was little awareness of how stakeholders could participate in local government activities. This finding contradicts that finding: There may not have been enough or any structures to let people know how to get involved in local government development activities.

It was crucial to ascertain whether the grassroots stakeholders' right to participate in the creation of the Assembly's MTDPs was actually exercised. Awareness of the process does not ensure effective participation since rational actors weigh costs and benefits before choosing a course of action [45]. The results on the degree of involvement, representation, and influence in the creation of MTDPs are in Table 4. Respondents rated the extent of involvement in MTDPs as very involved [55.4%], the level of representation of the local council in MTDPs as highly adequate [51.1%], and a modal group rated the level of influence in the design of MTDPs as highly influential [44%]. The results indicate that the stakeholders at the grassroots level were actively engaged in creating the MTDPs, that their representation from the area councils in the preparation of the MTDPs was also satisfactory, and that they had the authority to contribute to the MTDPs. It generally meets the NDPC's standards. Similarly, the key informants also indicated that during the development stage of the MTDP, they found that some of the residents, assembly members and area council members are typically involved. A typical situation was when a key informant from the Sawla-Tuna-Kalba District Assembly [June 30, 2022] remarked:

*During our last MTDP design, we had the involvement of the area council, assembly members, and residents participating in the process. They made valuable inputs in the deliberations*.

**Table 4:** Level of involvement, adequacy of representation and level of influence in the preparation of MTDP.

Variable	Response Category				Total
	Highly involved	Moderately involved	Slightly involved	Not at all involved	
Level of involvement	77 [55.4%]	44 [31.7%]	12 [8.6%]	6 [4.3%]	139 [100%]
	Highly adequate	Moderately adequate	Slightly adequate	Not at all adequate	
Adequacy in the representation of the area council	71 [51.1%]	43 [30.9%]	16 [11.5%]	9 [6.5%]	139 [100%]
	Highly influential	Moderately influential	Slightly influential	Not at all influential	
Level of influence	62 [44.6%]	50 [36.0%]	17 [12.2%]	10 [7.2%]	139 [00%]

The quotation hints that there is some level of grassroots involvement in the design of the MTDPs of the district assembly. As rational actors, the respondents actively participated because the advantages of their engagement outweighed the costs, which incentivised their participation [45,48]. Also, because grassroots stakeholders claimed that their level of influence in the production of MTDPs was considerable, their engagement may be classified as “citizen control,” which is the most significant level of participation according to Arnstein's ladder of citizen participation [40]. It exemplifies citizen power in which grassroots stakeholders may make decisions that are incorporated into MTDPs. Mohammad [37], Kobani [19], Rusnaini [21], and Agbenyo et al. [36] indicated that the participation of grassroots stakeholders in development planning within the local governance system was limited. Specifically, Rusnaini [21] observed that in Indonesia, participation in the Medium-Term Local Development Plan was at the placation level, which is a higher level of tokenism as stakeholders could provide advice.

Additionally, Spearman's Rank correlation was used to establish the relationship between MTDP awareness, involvement, and amount of influence. According to the test results [Table 5], all associations were statistically significant at the 1% level. Similarly, the positive correlation coefficients for the associations ranged from 0.401 to 0.573: While the association coefficients between awareness of MTDP and involvement were significant, the correlation coefficient for awareness as against representation adequacy and level of influence were medium. The results indicate that increased grassroots stakeholders' awareness of MTDPs favours their participation, representation, and influence.

**Table 5:** Spearman's ranks correlations matrix.

Variable	1	2	3	4	5	6	7
1. Awareness of MTDPs							
2. Involvement	.573*						
3. Satisfaction in preparation of MTDPs	.560*	.591*					
4. Capture Community needs	.435*	.540*	.715*				
5. Representation adequacy	.472*	.523*	.635*	.601*			
6. Satisfaction with the quality of participation at the area council	.405*	.405*	.565*	.589*	.514*		
7. Influence	.401*	.339*	.426*	.291*	.335*	.414*	
8. Level of confidence in the preparation of MTDP	.492*	.449*	.589*	.563*	.581*	.507*	.424*

Note: Correlation is significant at the 0.01* level [2-tailed]; n = 139; r = 0.10 to 0.29 or r = −0.10 to −0.29 small, r = 0.30 to 0.49 or r = −0.30 to −0.4.9 medium, r = 0.50 to 1.0 or r = −0.50 to −1.0 large.

Besides examining the level of participation, the study also investigated the degree to which grassroots stakeholders were satisfied and how confident they were that their participation in the preparation of the MTDPs would fulfil their community's needs. As shown in Table 6, most of the respondents [52.50%] were very satisfied with their level of participation in the development of the MTDP, and a similar proportion [51.80%] were very satisfied with the quality of their participation in the facilitation of meetings at the area council level. In addition, the majority of respondents were very confident or confident that their participation in the preparation of the MTDP satisfies the NDPC's requirements. Also, evidence from the key informant interviews showed that while some of the grassroots participants appeared satisfied with their involvement in the development of the MTDP, others were not, as they felt they did not have a voice. A typical situation was when it was stated:

*Some of the grassroots stakeholders have pointed out that they are confident that their contributions to the development of the MTDP will lead to their needs being met* [Key informant from the Sawla-Tuna Kalba District Assembly, June 30, 2022].

**Table 6:** Level of satisfaction and confidence in the preparation of the MTDP.

Variable	Response Category				Total
	Very Satisfied	Satisfied	Dissatisfied	Very Dissatisfied	
Level of satisfaction in the preparation of the MTDP	73 [52.5%]	42 [30.2%]	20 [14.4%]	4 [2.9%]	139 [100%]
Satisfied with the quality of the participation during the facilitation of meetings at the area council level	72 [51.8%]	42 [30.2%]	16 [11.5%]	9 [6.5%]	139 [100%]
	Very Confident	Confident	Unconfident	Very Unconfident	
Level of confidence in the preparation of MTDP	59 [42.4%]	56 [40.3%]	18 [12.9%]	6 [4.3%]	139 [100%]

The quote suggests that some of the grassroots stakeholders were satisfied with their participation as they felt it would translate into their felt needs. Arnstein's findings indicate that participation is the final rung on the ladder of citizen participation, where those involved have complete control over activities. Moreover, the satisfaction and confidence of grassroots stakeholders in their participation suggest that it can be categorised as self-mobilisation, according to Pretty's typology of participation [44], in which they can take independent initiatives. Moreover, a correlation analysis was conducted to determine the relationship between involvement in MTDP and satisfaction with the preparation of MTDPs, satisfaction with the quality of participation in the area council, and level of confidence in the preparation of MTDP, all of which were statistically significant with correlation coefficients ranging from 0.405 to 0.591 [Table 5]. It suggests that involvement in MTDPs positively affects the other variables.

### 4.3 Effects of participation in the MTDPs

**Fig. 5:**
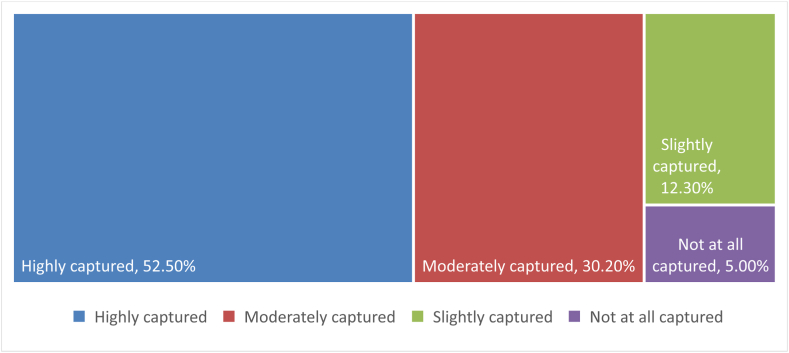
Level of the capture of community needs in the preparation of the MTDP.

Effective participation at the grassroots level in the development process ensures that their needs are met. This is the ultimate goal of all development efforts. Consequently, it was essential to determine whether or not grassroots participation in MTDPs promoted development. Fig. 5 shows that 52.5% of the respondents believed that their participation in the design of the MTDPs significantly contributed to capturing their community's developmental needs. Likewise, the interaction with the key informants revealed that the participation of the grassroots stakeholders had enabled the Assembly to provide for the needs of their residents.

The quotation indicates that involving the grassroots in the design of the MTDP assists in meeting their most pressing needs. The time spent designing the MTDP was worthwhile. It would motivate them to continue participating in future MTDP processes. Mubita et al. [38] concluded that stakeholder participation ensures that their needs are incorporated into development programmes and projects. According to the rational choice theory, participation enables grassroots stakeholders to meet their community's needs and incentivises them to participate in the development process. Aside from the fact that the respondents' needs appeared to have been met, this further supports Pretty's claim that their participation represents self-mobilisation. In addition, Spearman's rank correlation was used to determine the relationship between the capture of community needs in MTDP and participation in the preparation of MTDPs, satisfaction with the preparation of MTDPs, representation adequacy, and the ability to influence the preparation of MTDP. All the correlation coefficients were statistically significant, ranging between 0.291 and 0.601 [Table 5]. It implies that capturing community needs in MTDPs depends on participation, satisfaction, adequate representation, and influence in MTDP preparation.

### 4.4 Challenges to the implementation of the MTDPs

**Fig. 6:**
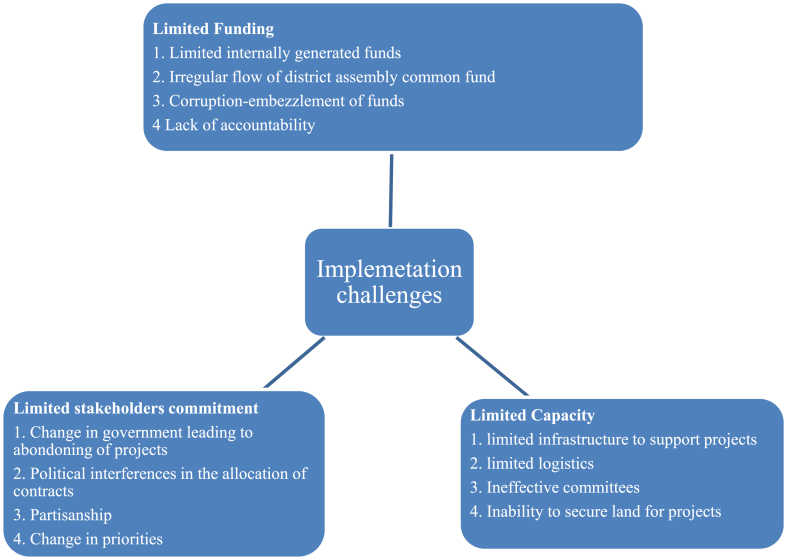
MTDP implementation challenges.

After developing the MTDP, the next step would be to implement it. The grassroots stakeholders would only be able to reap the benefits of their efforts following implementation. However, implementation is typically not without obstacles [18,37]. According to the available evidence, numerous obstacles hinder the implementation of the MTDP in the Sawla-Tuna-Kala District. These obstacles are broadly categorised into limited funding, stakeholder commitment, and capacity [Fig. 6]. These obstacles tend to impede the Assembly's ability to execute the MTDP that was designed. Consequently, grassroots participation becomes a waste of time. Participants lamented that during the area council meetings at Sawla:

*We spend our time contributing effectively to the development of this MTDP. I am, however, not sure what happens to its implementation. Previous plans have not been implemented adequately, and the Assembly keep complaining of no funds [Participant, Sawla, December 2021].*

Participants expressed this and several other similar views in the study. Management of the Sawla-Tuna-Kalba Assembly confirmed these assertions of participants but indicated that funds for the development of the Assembly are primarily from the central government, and releases of funds have been erratic over the years. Donor funds such as the District Development Fund [DDF] for district assemblies have also dwindled over the years, leaving the Assembly with limited funds to implement activities. Management of the Assembly, however, believed that if the citizen improved their payment of taxes, it would improve its internally generated funds [IGF] to support the implementation of planned activities.

Although some of the staff of the Assembly were cautious about citing specific cases, many of them confirmed the high level of political interference in implementing planned activities in the MTDP. They explained that some activities are priorities over others because of the political interest of leaders. The very pressing needs of some communities are sometimes ignored because those communities are not strongholds of the ruling political party. This situation becomes a real challenge in implementing MTDP as some community-level participants openly accused politicians of masterminding their communities' underdevelopment. Featuring very strong during interviews is the abandonment of projects after the change of government or the change of leadership of the Assembly. Participants cited examples of projects being abandoned in their communities, and Assembly kept indicating that those projects could not be continued because of no funds.

This circumstance can potentially discourage grassroots citizens from participating in MTDP activities. Consequently, MTDPs would not capture the felt needs of the populace, as they would withdraw from the process, and such projects would not be utilised by the populace, resulting in a waste of scarce resources. Related findings were made by Mohammad [37], Mensah [18], Odo [7], and Agbenyo et al. [23] that weak institutional structures, inadequate financial and human resources of the District Assemblies, land disputes, poor road networks, and low stakeholder commitment hindered the implementation of MTDPs in Bangladesh, Ghana and Nigeria. The challenges affecting the implementation of MTDPs appear not unique to Sawla-Tuna-Kalba District but are similar to other countries in the Global South. The existence of these challenges has the potential to limit the potential benefits that the people living within those local government areas can benefit from. As such, it will not motivate them to want to participate in the design of the MTDP since they know; eventually, it may not be implemented or will be partially implemented, which is tantamount to wasting their time.

## 5 Conclusions and policy implications

The study investigated the participation of grassroots stakeholders in MTDPs, its effects and the challenges of implementation of MTDPs. In general, grassroots stakeholders were aware of their eligibility to participate in the MTDPs' design. Many respondents believed that they were adequately represented in the design of the MTDPs, allowing them to wield influence over the process, demonstrating that awareness translated into actual participation. In addition, grassroots stakeholders were pleased and confident that their participation in developing MTDPs fulfilled community needs. However, obstacles such as limited funding, stakeholder commitment, and organisational capacity threaten the implementation of MTDPs, potentially limiting the benefits associated with grassroots stakeholders' participation. It was observed from the conclusions adduced here that the study's objectives were achieved. The strength of this study is that its results go against the common belief that stakeholders do not know they have the right to be involved in MTDPs, do not participate much, and when they do, they have no power. Nevertheless, the limitation of the study is that it used a cross-sectional study design which only provides the situation at one point in time: This design is not capable of providing a comprehensive picture of grassroots stakeholders' participation in terms of the previous situation and/or what will happen in the future. As such, any future related study could use longitudinal study designs to cure this shortfall.

Based on the findings, the Assembly must enhance its efforts to generate internal revenue. It can be accomplished by appealing to the residents to pay their levies and tolls. To complement this effort, revenue mobilisation regulations should be strictly enforced. To prevent officials from embezzling the collected funds, it will be necessary to implement accountability measures and controls. It will ensure that the revenue can be utilised to support the execution of the MTDPs. In addition, MTDPs should be depoliticised. The authorities in the Assembly should approach MTDPs objectively so that projects and programmes from them will continue under any government, thereby mitigating the threat of sunk costs. Lastly, the Assembly must ensure that the personnel and infrastructure capacities are improved. Regular in-service training will be required for the staff's capacity development to equip them with the appropriate skills and attitude for the job. As part of infrastructure development, the Assembly can lobby the central government and other development partners, such as non-governmental organisations, to provide them with some basic infrastructure to launch the MTDPs.

## Ethics statement

The ethics approval number is RERB 0020, issued by SD Dombo University of Business and Integrated Development Studies Research Ethics Review Board [RERB]. The researchers obtained the participants' informed consent through an informed consent letter.

## Author contribution statement

Asaah Sumaila Mohammed: Conceived and designed the analysis; Analyzed and interpreted the data.

Moses Naiim Fuseini: Conceived, Analyzed and interpreted the data; Wrote the paper.

Kuupiel Cuthbert Baba: Contributed analysis tools or data.

## Data availability statement

Data will be made available on request.

## Additional information

Supplementary content related to this article has been published online at [URL].

## Declaration of competing interest

The authors declare no conflict of interest.
