# Supplementary material for: Grassroots stakeholders’ perception of participation in the Medium-Term Development Plan of District Assemblies in Ghana: The case of Sawla-Tuna-Kalba District
Source: Heliyon. 2023 Aug 16;9(8):e19178. doi: 10.1016/j.heliyon.2023.e19178 (PMC10458331; doi:10.1016/j.heliyon.2023.e19178)
Supplement: Supplementary file 1 [file mmc1.docx]

**Appendix 1**

**Questionnaire for Grassroots Stakeholders**

**Dear Valued Respondent,**

The study is on *“Grassroots stakeholders’ perception of participation in the Medium-Term Development Plan of District Assemblies in Ghana: The case of Sawla-Tuna-Kalba District.”* Please note that whatever information you provide will be treated confidentially and used for only its intended purpose. Your voluntary participation is valuable. There are no risks involved in taking part in this study. As a voluntary participant, you are not required to answer any question that you do not wish to respond, and you can withdraw at any time during the process. Your answering of this questionnaire will indicate your consent to participate in this study.

**Instruction**

*Please, for each question, indicate the chosen option by ticking the most appropriate answer(s) and fill in (where applicable)*

1. What of this age categories do you belong?

01=21-40years

02=41-60

03=61+

1. What is your sex?

01=Male 02= Female

1. What is your educational level?

01= No education

02= Basic education

03= Secondary education

04= Tertiary education

1. How would you rate your level of awareness in the preparation of the Medium-Term Development Plan at the grassroots level as required by the National Development Planning Commission (NDPC) guidelines?

01= Highly aware

02= Moderately aware

03= Slightly aware

04= Not at all aware

1. How could you rate your level of involvement in the preparation of the Medium-term Development Plan (MTDP) at the grassroots level as required by the National Development Planning Commission (NDPC) guidelines?

01= Highly involved

02= Moderately involved

03= Slightly involved

04= Not at all involved

1. How would you rate your level of satisfaction in the preparation of the medium-Term Development Plan (MTDP) at the grassroots level as required by the national Development Planning Commission (NDPC) 'guidelines?

01= Very satisfied

02= Satisfied

03= Dissatisfied

04= Very dissatisfied

1. How would you rate the level of capture of community needs in the preparation of the Medium-Term Development Plan (MTDP) at the grassroots level as required by the National Development Planning Commission (NDPC) guidelines?

01= Highly captured

02= Moderately captured

03= Slightly captured

04= Not at all captured

1. How would you rate the level of adequacy in the representation of the area council in the preparation of the Medium-Term Development Plan (MTDP) at the grassroots level as required by National Development Planning Commission?

01= Highly adequate

02= Moderately adequate

03= Slightly adequate

04= Not at all adequate

1. To what extent are you satisfied with the quality of the participants during the facilitation of meetings at the area council level?

01= Very satisfied

02= Satisfied

03= Dissatisfied

04= Very dissatisfied

1. How would you rate your level of influence in the preparation of the Medium Term Development Plan (MTDP) at the grassroots level as required by the National Development Planning Commission (NDPC) guidelines?

01= Highly influential

02= Moderately influential

03= Slightly influential

04= Not at all influential

1. How would you rate your level of confidence in the preparation of the Medium Term Development Plan (MTDP) at the grassroots level as required by the National Development Planning Commission (NDPC) guidelines?

01= Very confident

02= Confident

03= Unconfident

04= Very unconfident

1. What challenges do you foresee challenging the assembly in the implementation of the activities outlined in the MTDP?

………………………………………………………………………………………………………………………………………………………………………………………………………………………………………………………………………………………………………………………………………………………………………………………………………………………………………………………………………………………………………………………………………………………………………………………………………………………………………………………………………………………………………………………………………………………………………………………………………………………………………………………………………..

Thank you for your participation

**Appendix 2**

**Interview Guide for Key Informants**

1. How would you describe the level of grassroots stakeholders’ awareness about the preparation of the Medium-Term Development Plan (MTDP)?
2. How would you describe the level of grassroots stakeholders’ involvement in the preparation of the MTDP at the grassroots level as required by the National Development Planning Commission (NDPC) guidelines?
3. How would you describe the level of grassroots stakeholders’ view of adequacy in their representation of the area council in the preparation of the MTDP?
4. How would you describe the level of grassroots stakeholders’ satisfaction with the quality of the participants during the facilitation of meetings at the area council level?
5. How would you describe the level of grassroots stakeholders’ level of influence in the preparation of the MTDP?
6. How would you describe the level of grassroots stakeholders’ level of confidence in the preparation of the MTDP?
7. How would you describe the level of grassroots stakeholders’ satisfaction in the preparation of the MTDP?
8. How would you describe the level of grassroots stakeholders’ view of the capture of community needs in the preparation of the MTDP?
9. What are the effects of grassroots stakeholders’ participation in the MTDP?
10. What challenges do you foresee challenging the assembly in the implementation of the activities outlined in the MTDP?

Thank you for your participation
